# Copper toxicity compromises root acquisition of nitrate in the high affinity range

**DOI:** 10.3389/fpls.2022.1034425

**Published:** 2023-01-20

**Authors:** Sebastian B. Feil, Monica Yorlady Alzate Zuluaga, Stefano Cesco, Youry Pii

**Affiliations:** Faculty of Science and Technology, Free University of Bozen/Bolzano, Bolzano, Italy

**Keywords:** copper, nitrate induction, cucumber, high affinity transport system, *NRT* genes, *PM H^+^-ATPase*, gene expression

## Abstract

The application of copper (Cu)-based fungicides for crop protection plans has led to a high accumulation of Cu in soils, especially in vineyards. Copper is indeed an essential micronutrient for plants, but relatively high concentrations in soil or other growth substrates may cause toxicity phenomena, such as alteration of the plant’s growth and disturbance in the acquisition of mineral nutrients. This last aspect might be particularly relevant in the case of nitrate $\left({\text{NO}}_{3^{-}}\right)$ , whose acquisition in plants is finely regulated through the transcriptional regulation of ${\text{NO}}_{3^{-}}$ transporters and plasma membrane H^+^-ATPase in response to the available concentration of the nutrient. In this study, cucumber plants were grown hydroponically and exposed to increasing concentrations of Cu (*i.e.*, 0.2, 5, 20, 30, and 50 µM) to investigate their ability to respond to and acquire ${\text{NO}}_{3^{-}}$ . To this end, the kinetics of substrate uptake and the transcriptional modulation of the molecular entities involved in the process have been assessed. Results showed that the inducibility of the high-affinity transport system was significantly affected by increasing Cu concentrations; at Cu levels higher than 20 µM, plants demonstrated either strongly reduced or abolished ${\text{NO}}_{3^{-}}$ uptake activity. Nevertheless, the transcriptional modulation of both the nitrate transporter *CsNRT2.1* and the accessory protein *CsNRT3.1* was not coherent with the hindered ${\text{NO}}_{3^{-}}$ uptake activity. On the contrary, *CsHA2* was downregulated, thus suggesting that a possible impairment in the generation of the proton gradient across the root PM could be the cause of the abolishment of ${\text{NO}}_{3^{-}}$ uptake.

## Introduction

Nitrogen (N) is a fundamental element for crops, and its limitations can severely affect plant development and productivity (Marschner, 2012). This nutrient can be acquired by roots from different sources, namely inorganic (e.g., nitrate— ${\text{NO}}_{3^{-}}$ and ammonia— ${\text{NH}}_{4^{+}}$ ) and organic (e.g., urea and amino acids) (Nacry et al., 2013; Zanin et al., 2015). However, among the inorganic N ones, ${\text{NO}}_{3^{-}}$ represents the main source for the majority of crops cultivated in aerobic agricultural soils (Crawford and Glass, 1998). The uptake kinetics of this form, when analyzed as a function of its external concentration, show a biphasic trend (Crawford and Glass, 1998; Forde and Clarkson, 1999), ascribable to at least two transport systems, the high and low affinity transport systems (HATS and LATS, respectively) (Touraine and Glass, 1997; Filleur et al., 2001). The HATS is generally more involved in the uptake when the external ${\text{NO}}_{3^{-}}$ concentration is lower than 0.5–1 mM, and it shows a saturable behavior, described by a Michaelis–Menten kinetic (Filleur et al., 2001). On the other hand, LATS is active when the ${\text{NO}}_{3^{-}}$ concentration in the growth medium is higher than 0.5–1 mM, and it displays non-saturable kinetics in which the uptake rate linearly increases along with the substrate concentration (Touraine and Glass, 1997). Moreover, ${\text{NO}}_{3^{-}}$ uptake at the level of the root plasma membrane (PM) is mediated by at least two gene families, *NRT1* and *NRT2* (Plett et al., 2010; Nacry et al., 2013). The *NRT1* gene encodes for low-affinity transporters, whereas the *NRT2* gene family encodes for high-affinity transporters (Plett et al., 2010). Furthermore, members of the NRT2 gene family are often expressed together with accessory proteins belonging to the NRT3 family, which are required for the functionality and regulation of HATS transporters (Zhou et al., 2000; Tong et al., 2005; Okamoto et al., 2006; Pii et al., 2016).

In field conditions, the ${\text{NO}}_{3^{-}}$ concentration in soil is variable in time and space. Thus, plants have adapted in order to modulate their ${\text{NO}}_{3^{-}}$ uptake capacity according to nutrient availability (Marschner, 2012). In fact, following the exposure of roots to ${\text{NO}}_{3^{-}}$ (like after nitrate fertilization of crops), an upregulation of genes encoding transporters belonging to the inducible HATS (iHATS) takes place (Siddiqi et al., 1989). This phenomenon is termed induction (Jackson et al., 1973), and it results in a higher ${\text{NO}}_{3^{-}}$ uptake rate by roots. The induction is a transient phenomenon, since the ${\text{NO}}_{3^{-}}$ uptake rate generally reaches its maximum in a few hours or days, in herbaceous species or tree plants, respectively (Crawford and Glass, 1998; Min et al., 1998; Pii et al., 2014), and it is afterwards downregulated, due to a negative feedback inhibition (Glass et al., 2001).

Nitrate uptake by roots is secondary active transport, therefore it requires metabolic energy to be sustained (Siddiqi et al., 1990; Glass et al., 1992). Previous evidence has demonstrated that ${\text{NO}}_{3^{-}}$ is taken up through a co-transporting mechanism along with H^+^, whereby the proton gradient across the PM is generated and maintained by the activity of the PM H^+^-ATPase (McClure et al., 1990a; McClure et al., 1990b; Glass et al., 1992; Santi et al., 1995). Several studies in the last years have shown that both the biochemical activity and the molecular regulation of PM H^+^-ATPase mirror the profile observed for the induction of ${\text{NO}}_{3^{-}}$ uptake rate (Santi et al., 2003; Nikolic et al., 2012; Pii et al., 2014; Pii et al., 2016), further disclosing the essential role of these molecular entities for HATS functionality. It is interesting to point out that PM H^+^-ATPase involved in the mineral acquisition processes in plants represents only a subgroup, namely P3 (Pedersen et al., 2012), of a wider gene family encompassing H^+^-ATPase enzymes featuring different physiological functions and substrate specificity (Axelsen and Palmgren, 1998; Palmgren and Nissen, 2011). Yet, despite the redundancy of PM H^+^-ATPase, only specific isoforms are involved in ${\text{NO}}_{3^{-}}$ uptake at the root level (Santi et al., 2003; Nikolic et al., 2012; Pii et al., 2014; Pii et al., 2016).

Copper (Cu) is an essential element for plants, being involved in a plethora of physiological functions such as, for instance, photosynthesis, respiration, C and N metabolism, and protection against oxidative stress (Marschner, 2012). Despite its crucial role in plants, it is required in very low amounts (Yruela, 2009). On the other hand, its excess might induce stressing conditions like alterations in photosynthesis capacity, stunted growth (Brunetto et al., 2016), root length reduction, and root tip darkening and thickening (Vinit-Dunand et al., 2002; Lequeux et al., 2010; Meier et al., 2012; Feigl et al., 2013). Moreover, when exposed to toxic Cu concentrations, plants can present nutritional disorders, displayed by an altered accumulation of macro- and micronutrients at both shoot and root levels (Baldi et al., 2018; Marastoni et al., 2019a; Marastoni et al., 2019b; Feil et al., 2020; Cesco et al., 2021). It has been recently demonstrated that a Cu excess can impair the ability of cucumber plants to take up phosphorus (P) from the growth medium by negatively affecting the expression of high-affinity transporters in roots (Feil et al., 2020). Similarly, Marastoni et al. (2019b) have recently shown that a Cu excess could differentially affect the expression of transporters involved in the uptake of micronutrients, whose extent was dependent on the tolerance level to the metal exhibited by the different grapevine rootstocks. Furthermore, previous pieces of research have indicated that Cu stress can alter N nutrition in plants, affecting both the ability of plants to take up ${\text{NO}}_{3^{-}}$ and ${\text{NH}}_{4^{+}}$ and the biochemical activities devoted to N assimilation (Xiong et al., 2006; Zhang et al., 2014; Hippler et al., 2018; Gong et al., 2019; Huo et al., 2020). In this context, it has been observed that prolonged Cu toxicity in *Arabidopsis thaliana* can downregulate the expression of *NRT1* genes encoding low-affinity nitrate transporters (*NRT1* family) as well as those encoding specific isoforms of the plasma membrane proton pump (*i.e.*, AHA2) (Hippler et al., 2018). On the contrary, medium Cu excess (*i.e.*, 5 and 10 µM) could induce the upregulation of the *NRT2* gene family in 24–76 h, albeit not at sufficient levels to restore N uptake at the root level. Despite these pieces of evidence, a detailed investigation concerning the Cu effects on ${\text{NO}}_{3^{-}}$ acquisition mechanisms has never been carried out so far.

Indeed, the increase in Cu concentration in agricultural soil is currently becoming a severe environmental problem, particularly in vineyards, mainly due to the application of cupric fungicides for crop defense plans (Cesco et al., 2021). In a context of greater sustainability in agriculture and the need to guarantee food for a growing population, it is therefore mandatory to deepen our knowledge about plant responses to Cu toxicity, particularly focusing on the dynamics of N acquisition. Despite N being a fundamental macronutrient for plants, research efforts to understand the relationships between Cu and N in this field are still very limited, and, to the best of our knowledge, the influence of Cu toxicity on the inducibility of ${\text{NO}}_{3^{-}}$ HATS genes has not been investigated yet. For these reasons, the main aim of our research was to investigate the ability of a crop model plant, *i.e.*, *Cucumis sativus* L., to respond to root application of ${\text{NO}}_{3^{-}}$ if exposed to increasing Cu concentrations. The kinetics of substrate uptake and the transcriptional modulation of the molecular entities involved in the process have been assessed.

## Material and methods

### Plant material and growing conditions

Cucumber (*C. sativus* L. cv. Chinese Long) seeds were germinated for 5 days in darkness at 22°C on filter paper moistened with 0.5 mM CaSO_4_ and placed vertically (Feil et al., 2020). Five-day-old seedlings were transferred into 2 L plastic pots filled with 1.5 L of a full-strength nutrient solution (NS) containing 2 mM Ca(NO_3_)_2_, 0.5 mM MgSO_4_, 0.7 mM K_2_SO_4_, 0.1 mM KCl, 10 µM H_3_BO_3_, 0.5 µM MnSO_4_, 0.2 µM CuSO_4_, 0.5 µM ZnSO_4_, 0.01 µM (NH_4_)_6_Mo_7_O_24_, 80 µM Fe-EDTA, buffered at pH 6 with 0.1 mM MES KOH, continuously aerated, and renewed twice a week (Marastoni et al., 2019a).

#### Experiment 1: Effect of Cu toxicity on NO_3^−^_ LATS and HATS

To investigate the effect of Cu toxicity on both the nitrate $\left({\text{NO}}_{3^{-}}\right)$ low affinity transport system (LATS) and the high affinity transport system (HATS), cucumber seedlings after germination were grown for 7 days in full-strength NS (see above). At the end of this cultivation period, plants were split into four groups and subjected to increasing concentrations of Cu (*i.e.*, 0.2, 5, 25, and 50 µM) for 7 days. At the end of the growing period, in the presence of different Cu concentrations, the ${\text{NO}}_{3^{-}}$ uptake rate was measured. Plants were removed from the NS, the root systems were washed in 0.1 mM CaSO_4_ solution for 5 min and then exposed for 7 min to an aerated uptake solution containing 1 mM MES-BTP, pH 6.0, and either 1 mM or 200 µM ^15^ ${\text{NO}}_{3^{-}}$ to test the low affinity or high affinity range, respectively. Three independent growth experiments were performed (three biological replicates). Each sample of each biological replicate consisted of roots pooled from two plants. The experiments were run in a climate chamber under controlled conditions (14/10 h light/dark, 24/19°C, 250 µmol m^−2^ s^−1^ light intensity, and 70% relative humidity).

#### Experiment 2: Effect of Cu toxicity on the induction of NO_3^−^_ HATS

For the HATS induction experiment, after germination, cucumber seedlings were transferred and grown for 7 days in full-strength NS (see above). At the end of this cultivation period, plants were then split into five groups and subjected to different Cu concentrations (*i.e.*, 0.2, 5, 20, 30, and 50 µM) for 7 days; the last 4 days of this second cultivation period, cucumber plants were also deprived of N (**Supplementary Figure 1**). The induction of HATS was carried out by treating N-starved plants with 250 µM Ca(NO_3_)_2_ (induced plants), whilst control plants were treated with 250 µM CaSO_4_ (not induced plants) (Pii et al., 2019), to balance the Ca^2+^ supplementation. To determine ${\text{NO}}_{3^{-}}$ uptake rate, plants were removed from the NS, the root systems were washed in 0.1 mM CaSO_4_ solution for 5 min and then exposed for 7 min to an aerated uptake solution containing 200 µM ^15^ ${\text{NO}}_{3^{-}}$ in 1 mM MES-BTP, pH 6.0 (see below). Nitrate uptake rate was measured 0, 4, 8, 12, and 24 h after the induction treatments (HAT). At each time point, plant roots were sampled and either dried to a constant weight for isotopic analyses or frozen in liquid N_2_ for gene expression analyses. Three independent growth experiments were performed (three biological replicates). Each sample of each biological replicate consisted of roots pooled from two plants. The experiments were run in a climate chamber under controlled conditions (14/10 h light/dark, 24/19°C, 250 µmol m^−2^ s^−1^ light intensity, and 70% relative humidity).

### Determination of nitrate uptake rate

Nitrate uptake assays were carried out using ^15^N-labeled calcium nitrate [Ca(^15^NO_3_)_2_, 60 atom% ^15^N, Sigma-Aldrich], as previously described (Pii et al., 2016; Pii et al., 2019). Briefly, the roots of each seedling were washed in 0.1 mM CaSO_4_ for 5 min and then exposed for 7 min to an aerated uptake solution composed of 100 μM Ca(^15^NO_3_)_2_ in 1 mM MES-BTP, pH 6.0. The roots were then rinsed for 2 min in 0.1 mM CaSO_4_ (Pii et al., 2016; Pii et al., 2019). Roots and shoots were then separated and dried at 70°C for 48 h until a constant weight was reached. The ^15^N content was determined using isotope ratio mass spectrometry analysis (Delta V isotope mass spectrometer, ThermoFisher Scientific), as previously described (Pii et al., 2019).

### Bioinformatics

The identification of *NRT2* and *NRT3* genes in the cucumber genome (using the Cucumber (Gy14) v2 genome, http://cucurbitgenomics.org/organism/16) was based on amino acid sequence similarity with NRT2 and NRT3 proteins of other organisms, such as *Vitis vinifera*, *A. thaliana*, *Populus trichocarpa*, *Glycine max*, *Solanum lycopersicum*, *Oryza sativa*, *Sorghum bicolor*, and *Zea mays* (Pii et al., 2014). Similarly, putative *PM H^+^-ATPase* genes were identified based on amino acid sequence similarity with PM H^+^-ATPase of *Nicotiana plumbaginifolia* Viv., *O. sativa* L., *A. thaliana* (L.) Heynh. (Arango et al., 2003), *V. vinifera* (Pii et al., 2014), and *Z. mays* (Pii et al., 2016). The amino acid sequences were obtained from public databases (http://www.ncbi.nlm.nih.gov/, http://www.uniprot.org/uniprot/). The predicted sequences for NRT2, NRT3, and PM H^+^-ATPase in cucumber were identified through a BLASTP search (Altschul et al., 1997). By using the selected putative proteins for NRT2, NRT3, and PM H^+^-ATPase, a phylogenetic analysis was performed; the amino acid sequences were aligned by the ClustalW algorithm (https://www.genome.jp/tools-bin/clustalw). Phylogenetic trees were built using the Phylogenetic Interference Package program (PHYLIP; University of Washington, http://evolution.genetics.washington.edu/phylip.html), and they were visualized through FigTree software version 1.4.4.

### Real-time quantitative (RT-qPCR) analysis

Total RNA was extracted from the stored frozen roots using the Spectrum Plant Total RNA Kit (Sigma-Aldrich) according to the manufacturer’s instructions. Afterwards, 1 μg of total RNA was treated with 10 U of DNAse RQ1 and used for cDNA synthesis by the ImProm-II Reverse Transcription System (Promega) and oligo(dT)_15_ primer as per the manufacturer’s recommendations. The quantitative real-time PCR reaction (qRT-PCR) was performed using the SsoFast EvaGreen Supermix (Bio-Rad). Gene-specific primers were designed for the target genes (**Supplementary Table 1**). Experiments were carried out in triplicate with the following conditions: 5 min at 95°C, followed by 40 cycles at 95°C for 30 s and 55°C for 30 s. The amplification efficiency was calculated from raw data using LinRegPCR software (Ramakers et al., 2003). For each transcript, the mean normalized expression value was calculated using the housekeeping transcript, and the relative expression ratio values were calculated by 2^−ΔΔCt^ method according to Livak and Schmittgen (2001).

### Statistical analyses

Data are presented as means ± SE, whereby n is represented by three independent biological replicates; each biological replicate consists of roots pooled from two plants. Depending on the dataset, the significance of differences among means was calculated by either Student’s t-test, one-way ANOVA with *post-hoc* Tukey HSD, or two-way ANOVA, as specified in figure legends. The statistical analyses and data visualization were carried out using R software v.3.6.1 and the packages listed in **Supplementary Information 1**.

## Results

### Effect of Cu toxicity on nitrate uptake (Experiment 1)

**Figure 1:**
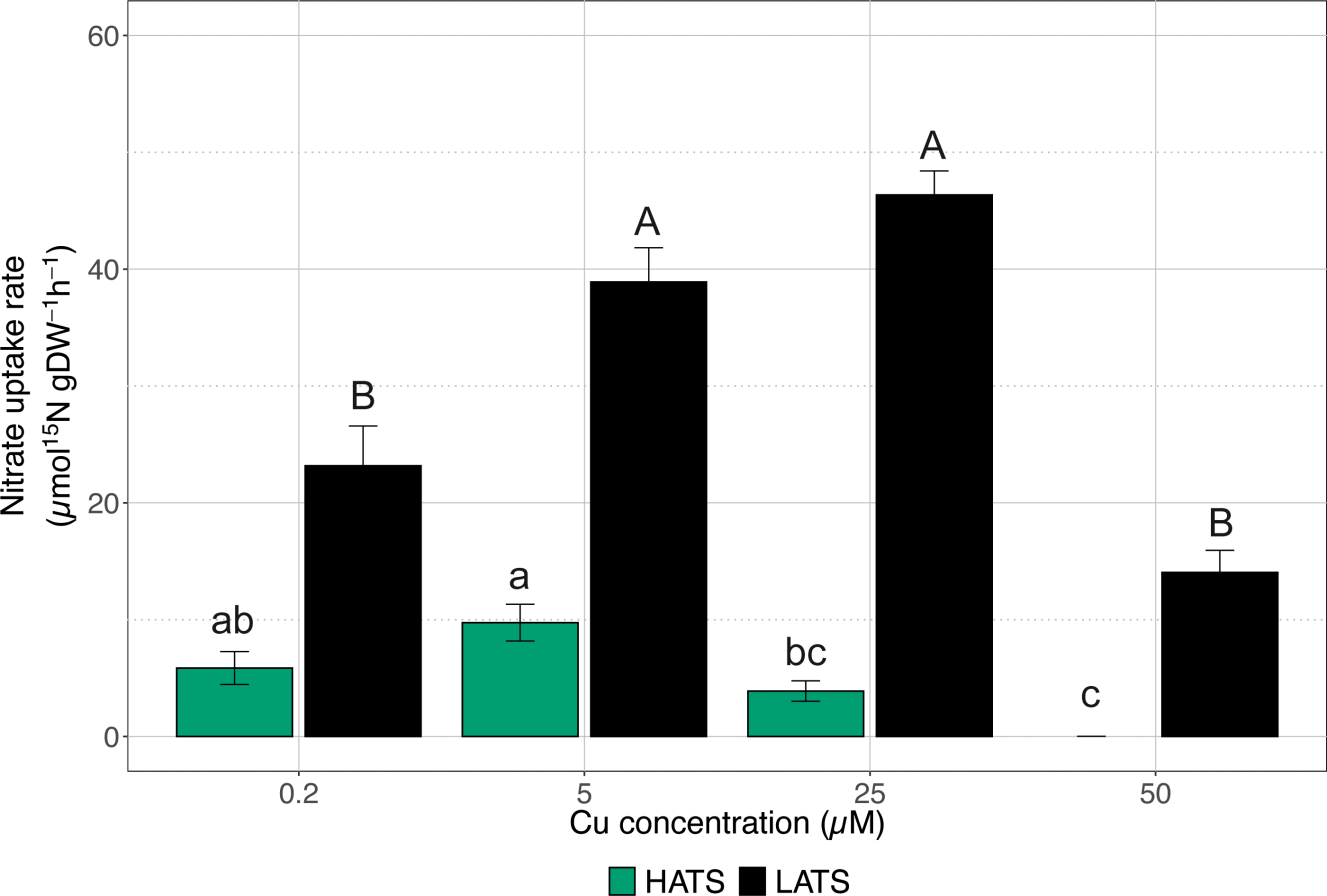
High-affinity (HATS) and low-affinity (LATS) ${\text{NO}}_{3^{-}}$ uptake rates in the roots of cucumber plants. Cucumber plants have been exposed to increasing concentrations of Cu (0.2, 5, 25, 50 µM). Uptake rates were determined by placing the seedlings in either 200 μM or 1 mM ^15^ ${\text{NO}}_{3^{-}}$ solution for 7 min in HATS and LATS, respectively. Data are the means ± SE of three independent biological replicates; each biological replicate was obtained by pooling two independent plants. Different letters indicate significantly different values as determined using a one-way ANOVA with Tukey post hoc tests (P<0.001).

The effects of Cu toxicity on both nitrate HATS and LATS transporters were investigated by exposing plants to increasing Cu concentrations (*i.e.*, 0.2, 5, 25, and 50 µM). As shown in **Figure 1**, the heavy metal did not negatively affect the ${\text{NO}}_{3^{-}}$ uptake rate in the low affinity range; when plants were exposed to 50 µM Cu, the activity of LATS did not show a significant difference compared to control plants (*i.e.*, plants treated with 0.2 µM Cu). On the other hand, cucumber plants treated with 5 and 25 µM Cu displayed a ${\text{NO}}_{3^{-}}$ uptake rate that was significantly higher as compared to control samples (**Figure 1**). As far as HATS is concerned, the highest Cu concentration (*i.e.*, 50 µM) completely abolished the ability of cucumber plants to acquire ${\text{NO}}_{3^{-}}$ (**Figure 1**). The other Cu levels tested did not significantly affect the ${\text{NO}}_{3^{-}}$ uptake rate compared to control samples, although starting at 5 µM Cu, a decreasing trend was observed (**Figure 1**).

### Nitrate induction is prevented by high Cu availability (Experiment 2)

**Figure 2:**
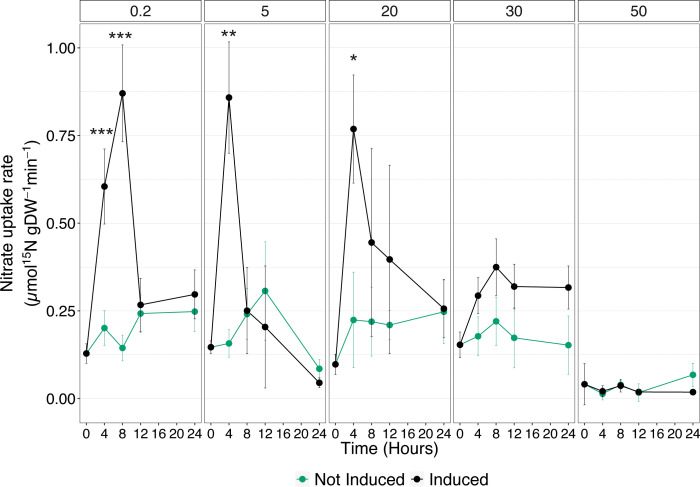
High-affinity ${\text{NO}}_{3^{-}}$ uptake rates in the roots of cucumber plants. Plants assessed have been either induced or not induced, and exposed to increasing concentrations of Cu (0.2, 5, 20, 30, and 50 µM). Uptake rates were determined by placing the seedlings in 200 μM ^15^ ${\text{NO}}_{3^{-}}$ solution for 7 min. Data are the means ± SE of three independent biological replicates; each biological replicate was obtained by pooling two independent plants. The statistical significance at each time point was determined using Student’s t-test: *, P<0.05; **, P<0.01; ***, P<0.001.

To deepen the understanding of Cu effects on the high-affinity ${\text{NO}}_{3^{-}}$ uptake system, ${\text{NO}}_{3^{-}}$ acquisition rates were measured in cucumber seedlings, either not-induced or induced with 500 µM ${\text{NO}}_{3^{-}}$ , at different times (0, 4, 8, 12, and 24 HAT) that were previously exposed to increasing Cu concentrations (*i.e.*, 0.2, 5, 20, 30, and 50 µM) (**Supplementary Figure 1**). In control plants, the ${\text{NO}}_{3^{-}}$ uptake rate showed the expected behavior following treatment with ${\text{NO}}_{3^{-}}$ . Induced plants displayed a significant increase in the nutrient uptake velocity already at 4 HAT and reached its maximum at 8 HAT, as compared to not induced seedlings. Afterwards, the ${\text{NO}}_{3^{-}}$ uptake rate declined, and at 12 and 24 HAT, it was comparable to the values obtained in not-induced plants (**Figure 2**). In plants exposed to 5 and 20 µM Cu, the induction with ${\text{NO}}_{3^{-}}$ caused an increase in the nutrient uptake rate, which reaches its highest value at 4 HAT and declines soon after to rate values comparable to those of not-induced plants (**Figure 2**). Interestingly, the maximum levels of uptake rates obtained in induced plants exposed to 5 and 20 µM Cu are comparable to those observed in control samples. On the other hand, when the Cu concentration was increased to 30 µM, the enhancement in ${\text{NO}}_{3^{-}}$ uptake rate following induction was completely abolished. Nevertheless, the constitutive uptake activity of not-induced plants was maintained to the same extent with respect to induced plants exposed to either 0.2, 5, or 20 µM Cu (**Figure 2**). Consistently with the results obtained in the previous experiment (**Figure 1**), 50 µM Cu in the nutrient solution not only prevented the upregulation of ${\text{NO}}_{3^{-}}$ uptake rate following induction, but it also abolished the ability of cucumber plants to take up ${\text{NO}}_{3^{-}}$ from the growth substrate (**Figure 2**).

### Bioinformatics

With the aim of gaining a deeper insight into the influence of increasing concentrations of Cu on the molecular mechanisms involved in ${\text{NO}}_{3^{-}}$ uptake in cucumber plants, the transcriptional modulation of the molecular entities involved in the response to ${\text{NO}}_{3^{-}}$ induction, namely *NRT2*, *NRT3*, and *PM H^+^-ATPase* genes, has been investigated.

Genes encoding for putative NRT2 and NRT3 proteins in the *C. sativus* genome were identified on the basis of protein sequence homology with members of the NRT2 family of *V. vinifera*, *A. thaliana*, *P. trichocarpa*, *G. max*, *S. lycopersicum*, *O. sativa*, *S. bicolor*, and *Z. mays* (Pii et al., 2014). This approach allowed the identification of two proteins (Cucsa.268720.1 and Cucsa.286270.1) encoding for putative high affinity ${\text{NO}}_{3^{-}}$ transporters; the phylogenetic analysis showed that both clustered with previously characterized high affinity transporters belonging to other dicot species (**Supplementary Figure 2**). In particular, Cucsa.268720.1, hereafter referred to as CsNRT2.1, clustered in a branch of the phylogenetic tree formed by different transporters isolated from *P. trichocarpa*, *V. vinifera*, and *G. max* genomes, yet displaying a higher degree of homology with GmNRT2.1, whereas Cucsa.286270.1, hereafter referred to as CsNRT2.5, showed the highest homology with NRT2.5 transporters of both dicots and monocots (**Supplementary Figure 2**). The same approach allowed the isolation of a single protein, Cucsa.098830.1, encoding a putative NRT3 orthologous gene in the cucumber genome. The phylogenetic analysis showed that Cucsa.098830.1 clustered together with the other proteins belonging to dicots and featuring a higher degree of homology with LjNAR2.1 (**Supplementary Figure 3**).

The identification of putative PM H^+^-ATPase has been achieved as previously described (Pii et al., 2016). The Blastp analysis carried out on *C. sativus* genome allowed the retrieval of 10 sequences putatively encoding PM H^+^-ATPase, whereas the phylogenetic analysis showed that three sequences (Cucsa,158480.1, Cucsa.197480.1, and Cucsa.097990.1) belong to subfamily I, four sequences (Cucsa.311000.1, Cucsa.161790.1, Cucsa.081200.1, and Cucsa.089200.1) to subfamily II and three sequences (Cucsa.132660.1, Cucsa.350780.1, and Cucsa.096210.1) to subfamily IV (**Supplementary Figure 4**).

### Gene expression analyses

**Figure 3:**
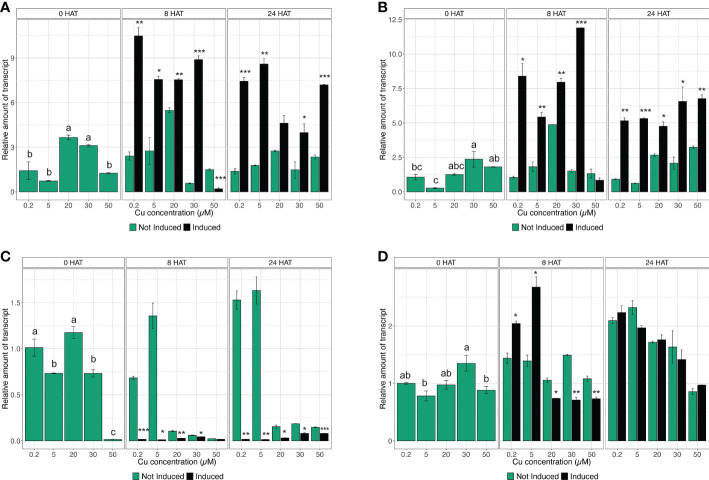
Time-course expression analysis of *CsNRT2.1*, *CsNRT3.1*, *CsNRT2.3*, and *CsHA2.* The expression levels of *CsNRT2.1* **(A)**, *CsNRT3.1* **(B)**, *CsNRT2.5* **(C)**, and *CsHA2* **(D)** were assessed by qRT-PCR in the roots of cucumber plants, either not-induced or induced with ${\text{NO}}_{3^{-}}$ and subjected to increasing concentration of Cu (0.2, 5, 20, 30, and 50 µM), at 0, 8, and 24 HAT. The data are normalized to the *translation elongation factor isoform 1-alpha*. The relative expression ratios were calculated using not induced control roots sampled before the treatments (not induced, 0.2 µM Cu at 0 HAT). Data are means (±SE), n = 3. Different letters within a time-point indicate significantly different values as determined using one-way ANOVA with Tukey *post hoc* tests (P<0.001). The statistical significance between not-induced and induced samples was tested by Student’s *t*-test: *, P<0.05; **, P<0.01; ***, P<0.001).

The quantification of gene expression has been carried out at key time points (*i.e.*, 0, 8, and 24 HAT) during the induction experiment, in accordance with the results obtained in the assessment of ${\text{NO}}_{3^{-}}$ uptake rate. Considering the high-affinity ${\text{NO}}_{3^{-}}$ transporters, the gene expression analysis confirmed that *CsNRT2.1* in control plants (i.e., 0.2 µM Cu) was modulated as expected (**Figure 3A**). In fact, *CsNRT2.1* did not show any alteration in the mRNA abundance in not induced plants throughout the time-course experiment, whilst in induced plants it presented a significant induction at 8 HAT and a reduction in the expression levels at 24 HAT, indeed mirroring ${\text{NO}}_{3^{-}}$ uptake rate data (**Figure 3A**). Similar responses have also been observed in plants exposed to 20 and 30 µM Cu (**Figure 3A**). On the other hand, in plants treated with 5 µM Cu, ${\text{NO}}_{3^{-}}$ induction significantly upregulated *CsNRT2.1* expression at 8 HAT, and it was maintained at the same levels until 24 HAT (**Figure 3A**). When plants were exposed to 50 µM Cu, *CsNRT2.1* was downregulated at 8 HAT and upregulated at 24 HAT with respect to not-induced cucumber plants (**Figure 3A**). Interestingly, the gene *CsNRT3.1* encoding the accessory protein displayed very similar transcriptional regulation as compared to *CsNRT2.1* (**Figure 3B**), confirming the coordinated activity of these two molecular entities in the induction of ${\text{NO}}_{3^{-}}$ uptake in plants (Pii et al., 2016).

The qRT-PCR analysis carried out on *CsNRT2.3* showed that, in control plants (*i.e.*, 0.2 µM Cu) and in plants treated with 5 µM Cu, the gene did not respond to induction with ${\text{NO}}_{3^{-}}$ , yet showed a downregulation at both 8 and 24 HAT in comparison to not-induced plants (**Figure 3C**). As far as 20 and 30 µM Cu-treated plants are concerned, *CsNRT2.3* was downregulated in not-induced and induced plants at both 8 and 24 HAT with respect to plants at 0 HAT (**Figure 3C**). At a concentration of 50 µM Cu, *CsNRT2.3* always resulted in downregulation to control plants at 0 HAT (**Figure 3C**).

After a preliminary analysis, only four of the 10 genes putatively encoding PM H^+^-ATPase, namely *Cucsa.089200.1* (already known as *CsHA2*), *Cucsa.081200.1* (already known as *CsHA3*), *Cucsa.161790.1*, and *Cucsa.311000.1*, were shown to be expressed in the root tissue of cucumber plants. Nonetheless, the qRT-PCR analyses highlighted that only *CsHA2* was modulated during ${\text{NO}}_{3^{-}}$ uptake induction (**Figure 3D** and data not shown). The assessment of gene expression levels allowed demonstrating that, before induction (*i.e.*, 0 HAT), the different Cu concentrations applied to cucumber plants did not affect the modulation of *CsHA2*, as it was not altered as compared to control plants (**Figure 3D**). The treatment with ${\text{NO}}_{3^{-}}$ caused in both 0.2 and 5 µM Cu-supplemented plants a significant upregulation in *CsHA2* at 8 HAT, which is consistent with the induction observed in both *CsNRT2.1*, *CsNRT3.1*, and the ${\text{NO}}_{3^{-}}$ uptake rate (**Figures 2**, **3**). On the contrary, at higher Cu concentrations, namely 20, 30, and 50 µM, the expression levels of *CsHA2* in induced plants were lower than those of not induced plants at 8 HAT and not significantly modulated at 24 HAT (**Figure 3D**).

## Discussion

In the last few years, the relationship between soil contamination with heavy metals and the responses of agricultural crops exposed to such stressing conditions has been investigated from different points of view. The accumulation of Cu in soils often represents a consequence of anthropogenic activities, among which is the intensive use of cupric fungicides for the defense of fruit crops (Rooney et al., 2006; Brunetto et al., 2016; Hippler et al., 2016; Cesco et al., 2021). It is estimated that the average Cu concentration of the Earth’s crust ranges between 6 and 80 mg kg^−1^ (Brunetto et al., 2016); nevertheless, the distribution of Cu between the solid phase and the soil solution is dependent on the physical and chemical properties of soils, which regulate precipitation/dissolution, adsorption/desorption, and redox reactions (Brunetto et al., 2016). On these bases, the concentration of Cu in the soil solution of arable soils lies between 15 nM and 5 µM (Cesco et al., 2021). However, several surveys indicate that Cu concentrations in the superficial soil horizon can often reach 200 mg kg^−1^, depending on anthropogenic activities, and sometimes exceed 1,000–3,000 mg kg^−1^ in contaminated soil (Flores-Vélez et al., 1996; Mirlean et al., 2007), suggesting that the bioavailable Cu concentrations may be significantly higher than 5 µM. Despite being an essential plant micronutrient (Yruela, 2009), relatively high concentrations of Cu in the growth substrate can induce toxicity phenomena in plants, which are generally shown through reduced vegetative growth, the alteration of biochemical processes (e.g., photosynthesis, lipid peroxidation), and an imbalanced acquisition of mineral nutrients (Baldi et al., 2018; Marastoni et al., 2019a; Marastoni et al., 2019b; Feil et al., 2020). Previous pieces of evidence have highlighted that different plant species grown on substrates featuring medium-high (e.g., 0.2 to 20 µM) Cu concentrations were impaired in their ability to acquire N, either ${\text{NO}}_{3^{-}}$ or ${\text{NH}}_{4^{+}}$ , and to assimilate it (Xiong et al., 2006; Zhang et al., 2014; Hippler et al., 2018; Gong et al., 2019; Huo et al., 2020). In particular, Cu toxicity was shown, over the long term, to affect the transcriptional modulation of genes encoding both high affinity and low affinity transporters and to hinder the activity of nitrated reductase (NR) and nitrite reductase (NiR), responsible for the first steps of nitrate assimilation (Xiong et al., 2006; Zhang et al., 2014; Hippler et al., 2018; Gong et al., 2019; Huo et al., 2020). In any case, it should be highlighted that a certain degree of variation in the responses has been observed in the different plant species investigated.

In cucumber plants, exposure over a long period to increasing Cu concentrations (at least up to 25 µM) apparently has a promoting effect on the activity of the LATS transport system (**Figure 1**), even though the highest concentration tested (*i.e.*, 50 µM) had a detrimental effect on the nitrate uptake rate (**Figure 1**). These observations are in agreement with the findings of Hippler et al. (2018), according to which the exposure of *A. thaliana* plants to medium-high concentrations of Cu could induce significant downregulation of genes belonging to the *NRT1* family. On the other hand, the same authors showed that Cu toxicity could increase the expression of genes belonging to the *NRT2* family, although ${\text{NO}}_{3^{-}}$ uptake was prevented over the period of time considered (Hippler et al., 2018). Indeed, these observations support our results, according to which ${\text{NO}}_{3^{-}}$ uptake in the high affinity range was depressed by Cu concentrations higher than 25 µM (**Figure 1**). However, in natural conditions, the availability of ${\text{NO}}_{3^{-}}$ in the growth substrate is subject to fluctuations, so plants had to adapt by evolving the induction phenomenon to maximize their ability to acquire the nutrient when it was available (Jackson et al., 1973). However, regardless of the importance of this aspect for plant nutrition, to the best of our knowledge, investigations about the influence of Cu toxicity on the inducibility of the HATS transport system in crop plants were lacking.

As widely assessed in previous studies (Kronzucker et al., 1995; Pii et al., 2014; Pii et al., 2016; Pii et al., 2019), the induction phenomenon caused a transient increase in the ${\text{NO}}_{3^{-}}$ uptake rate in roots of control plants (*i.e.*, 0.2 µM Cu), reaching the highest values at 8 HAT and showing afterwards a decrease back towards values close to those of not induced plants (**Figure 2**). The observed behavior in the ${\text{NO}}_{3^{-}}$ uptake dynamics is determined by the transcriptional modulation of high-affinity ${\text{NO}}_{3^{-}}$ transporter genes (*NRT2* gene family), the accessory protein (NRT3), and plasma membrane (PM) H^+^-ATPases (McClure et al., 1990a; McClure et al., 1990b; Glass et al., 1992; Santi et al., 1995; Pii et al., 2014; Pii et al., 2016; Pii et al., 2019). As expected, the qRT-PCR analyses confirmed that in control plants the increase in the ${\text{NO}}_{3^{-}}$ uptake rate was sustained by an upregulation at 8 HAT of the genes involved in the process, particularly *CsNRT2.1*, *CsNRT3.1* and *CsHA2*, encoding for a nitrate transporter, the accessory protein, and a PM H^+^-ATPase, respectively, while a negative feedback regulation was detected at 24 HAT (**Figure 3**). On the other hand, qRT-PCR studies also highlighted that *CsNRT2.3* was not involved in the response to ${\text{NO}}_{3^{-}}$ induction, at least in our experimental conditions (**Figure 3**).

With increasing Cu concentrations (*i.e.*, 5 and 20 µM) in the growth medium, the peak of ${\text{NO}}_{3^{-}}$ uptake rate due to induction was anticipated at 4 HAT, followed by a steep downregulation of the phenomenon (**Figure 2**). Nevertheless, the detected values of ${\text{NO}}_{3^{-}}$ uptake rate were comparable to those obtained in induced plants exposed to physiological levels of Cu. On the contrary, supplementation with higher concentrations of Cu (*i.e.*, 30 and 50 µM) completely abolished the plant response to induction, as if they were insensitive to the presence of ${\text{NO}}_{3^{-}}$ in the external medium (**Figure 2**). Interestingly, a similar effect has also been observed by Rizzardo and co-workers (2012), who demonstrated an impairment in ${\text{NO}}_{3^{-}}$ induction in maize plants treated with 10 µM Cadmium (Cd). However, the impairment in ${\text{NO}}_{3^{-}}$ induction observed in 30 and 50 µM Cu-treated plants was not mirrored by the transcriptional modulation of genes. In fact, in plants grown at 30 µM Cu, *CsNRT2.1* and *CsNRT3.1* were strongly upregulated in induced plants with respect to not-induced ones at both 8 and 24 HAT (**Figure 3**), thus suggesting that the ability of plants to sense ${\text{NO}}_{3^{-}}$ in the external medium was not compromised in these conditions. On the other hand, when plants were exposed to the highest Cu concentration (*i.e.*, 50 µM), *CsNRT2.1* and *CsNRT3.1* showed a delayed upregulation in induced plants, being in fact significantly more expressed only at 24 HAT (**Figure 3**); however, such transcriptional regulation did not correspond to a significant increase in the ${\text{NO}}_{3^{-}}$ uptake rate in induced plants (**Figure 2**).

The plasma membrane (PM) of epidermal root cells represents the first cellular structure that is the target for heavy metal toxicity in soil, which is well known to cause membrane lipid damage and alter the ionic homeostasis capacity of cells (Fodor et al., 1995). Considering that the activity of PM H^+^-ATPases is at the base of the electrochemical gradient across the membrane energizing the nutrient acquisition from the soil solution, it is clear that the regulation and functionality of these enzymes might be particularly crucial for plants exposed to heavy metal toxicity (Pedersen et al., 2012). In our experimental model, when plants were treated with 5 µM Cu, *CsHA2* was upregulated at 8 HAT in response to ${\text{NO}}_{3^{-}}$ induction, as in induced samples not treated with excessive amounts of Cu (**Figure 3**). Notwithstanding, *CsHA2* induction levels in 5 µM Cu-treated plants were higher compared to reference samples (*i.e.*, induced plants treated with 0.2 µM Cu), as though the slightly higher heavy metal concentration in the growth medium might require a further contribution of PM H^+^-ATPase to maintain the root cell ion homeostasis. Indeed, previous studies have demonstrated that cucumber roots exposed to 10 µM Cu or Cd for a short period of time (*i.e.*, 6 days) showed a higher expression of different *CsHA* isoforms and an increased proton pumping activity, further corroborating the role of these enzymes in the response to enhanced concentrations of heavy metals in the growth medium (Janicka-Russak et al., 2008; Janicka-Russak et al., 2012). The exposure to higher concentrations of Cu (20, 30, and 50 µM) compromised the responsiveness of *CsHA2* (**Figure 3**). At 8 HAT, the gene was in fact downregulated in ${\text{NO}}_{3^{-}}$ treated plants as compared to not-induced ones, while the expression levels were not significantly modulated in plants analyzed at 24 HAT (**Figure 3**). It is worth noting that loss in the responsiveness of PM H^+^-ATPase transcriptional regulation, which is normally observed during the induction (Santi et al., 2003; Nikolic et al., 2012; Pii et al., 2016), is coherent with the impaired ${\text{NO}}_{3^{-}}$ uptake in plants treated with higher Cu concentrations (**Figure 2**). However, information concerning the transcriptional regulation of *CsHA* following the exposure to toxic concentration of Cu is very little. Janicka-Russak et al. (2008), by applying a semi-quantitative RT-PCR approach, showed that *CsHA3* transcript abundance was not significantly affected by exposing plants to 100 µM Cu for 2 h. Later, similar results were obtained *via* quantitative RT-PCR analyses carried out on different isoforms of *CsHA* isolated from cucumber plants exposed to 10 µM Cu for 6 days, also demonstrating no influence of Cu toxicity on PM H^+^-ATPase enzyme abundance (Janicka-Russak et al., 2012). Despite this, it has been shown that the transcriptional regulation of PM H^+^-ATPase can be negatively affected in maize plants treated with 10 µM Cd, further suggesting a possible interference of heavy metals in these mechanisms and also considering the demonstrated impact on HATS induction (Rizzardo et al., 2012). On the other hand, an inhibitory effect due to short-term exposure to Cu on PM H^+^-ATPase functionality was demonstrated in the roots of different plant species (Kennedy and Gonsalves, 1989; Fodor et al., 1995). More recently, Astolfi and co-workers (2003; 2005) reported inhibition of activity of PM H^+^-ATPase in oat and maize roots after long-term treatment (7 and 21 days) with Cd.

## Conclusions

This research study was aimed at understanding the effects of excessive Cu availability in the rhizosphere of *C. sativus* L. plants on the biochemical and molecular mechanisms devoted to the acquisition of ${\text{NO}}_{3^{-}}$ , particularly focusing on the induction of the high affinity transport system (HATS). When the ${\text{NO}}_{3^{-}}$ concentration in the substrate was higher than 1 mM, Cu did not show any negative influence on nutrient uptake but rather promoted it at lower Cu concentrations. On the contrary, the inducibility of HATS was significantly affected by increasing Cu concentrations, which indeed showed a detrimental impact by preventing cucumber plants from the uptake of ${\text{NO}}_{3^{-}}$ . Especially in the cases of Cu concentrations higher than 20 µM, cucumber plants demonstrated either a strongly reduced or abolished ${\text{NO}}_{3^{-}}$ uptake activity, albeit the transcriptional modulation of both the nitrate transporter *CsNRT2.1* and the accessory protein *CsNRT3.1* was coherent with the expected induction. Nevertheless, in these conditions, genes encoding PM H^+^-ATPase (*i.e.*, *CsHA2*) were downregulated instead of induced, thus suggesting that a possible impairment in the generation of the proton gradient across the root PM could be the cause of the abolishment of ${\text{NO}}_{3^{-}}$ uptake.

These findings are particularly relevant considering that, as mentioned above, the bioavailable Cu concentration in contaminated soils may exceed 5 µM level that has been pointed out as a threshold for toxicity, above which also a severe impairment in the uptake of N and P (Feil et al., 2020) has been demonstrated. Despite the need for additional investigation to better understand the molecular bases underpinning the interaction between Cu and the transcriptional regulation of PM H^+^-ATPase, the data presented further highlight that increasing concentrations of this heavy metal in agricultural soils can represent an issue to be addressed in the near future, not only for its documented toxic effects on crops but also for its ability to inhibit or impair the uptake of macro and micronutrients by other plants, consequently impacting on the efficiency and sustainability of the agricultural practice of crop fertilization with nitrate.

## Data availability statement

The original contributions presented in the study are included in the article/**Supplementary Material**. Further inquiries can be directed to the corresponding author.

## Author contributions

Experimental Design: SC and YP. Experiments execution and data collection: SF and MA. Data analyses and visualization: MA and YP. Data interpretation: MA, SC, and YP. Manuscript writing and critical revision: SF, MA, SC, and YP. Financial support: YP. All authors contributed to the article and approved the submitted version.
