# Supplementary material for: Copper toxicity compromises root acquisition of nitrate in the high affinity range
Source: Front Plant Sci. 2023 Jan 20;13:1034425. doi: 10.3389/fpls.2022.1034425 (PMC9895927; doi:10.3389/fpls.2022.1034425)
Supplement: Supplementary file 1 [file DataSheet_1.docx]

R version 3.6.1 (2019-07-05)

Platform: x86_64-apple-darwin15.6.0 (64-bit)

Running under: macOS 10.16

Matrix products: default

LAPACK: /Library/Frameworks/R.framework/Versions/3.6/Resources/lib/libRlapack.dylib

Packages:

- ggbreak_0.0.7
- agricolae_1.3-1
- plyr_1.8.4
- ggplot2_3.3.5
