# Supplementary figures and images for: Copper toxicity compromises root acquisition of nitrate in the high affinity range
Source: Front Plant Sci. 2023 Jan 20;13:1034425. doi: 10.3389/fpls.2022.1034425 (PMC9895927; doi:10.3389/fpls.2022.1034425)

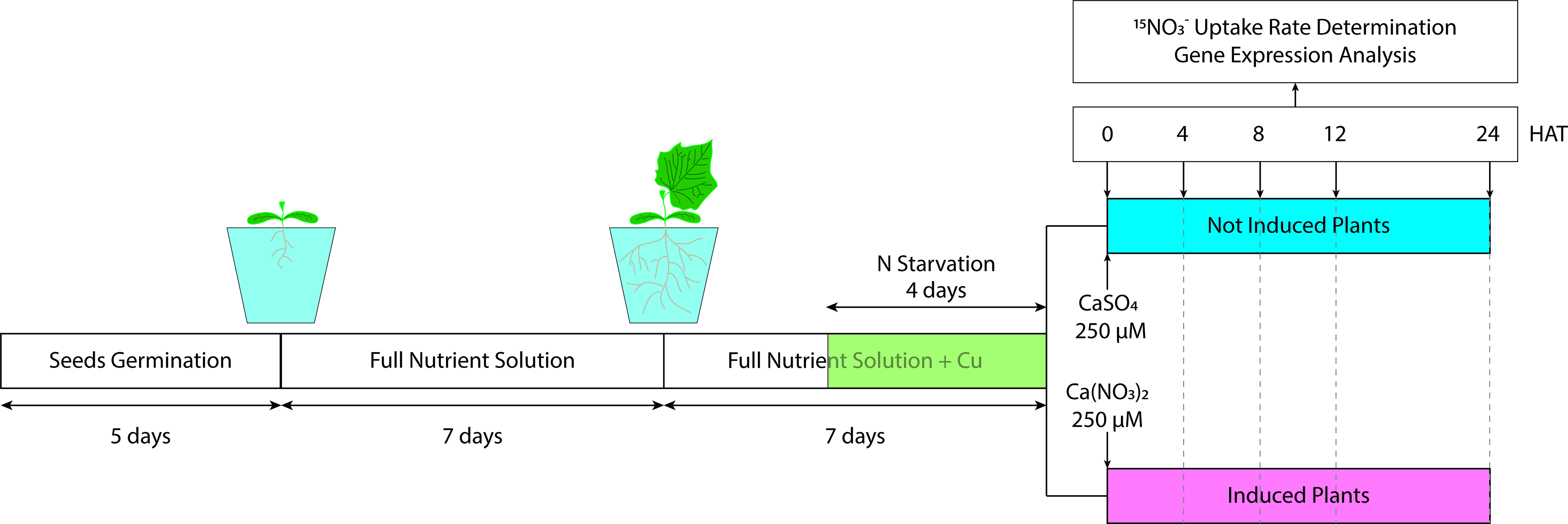

Supplement: Supplementary file 2 [file Image_1.tif]

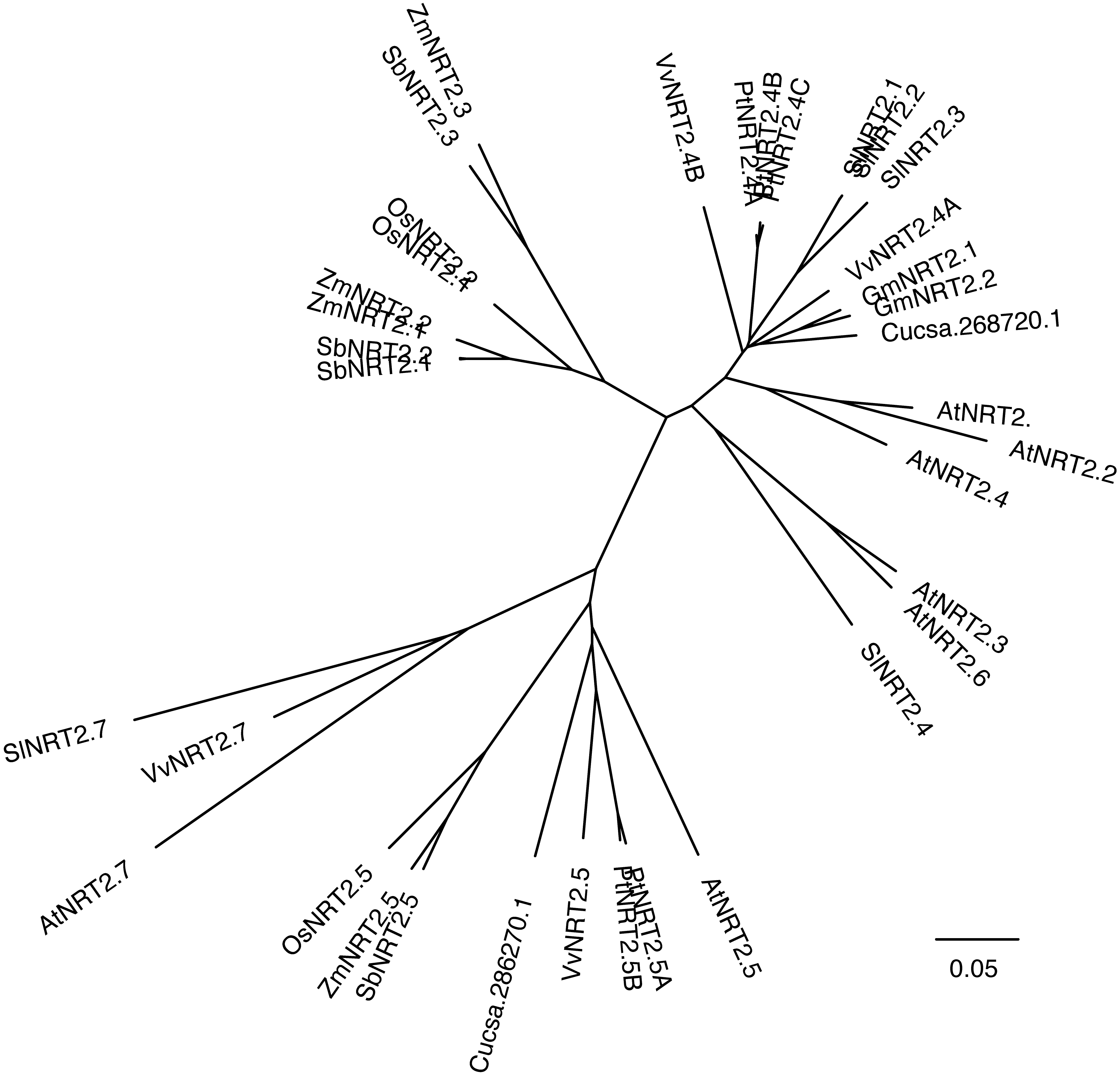

Supplement: Supplementary file 3 [file Image_2.tif]

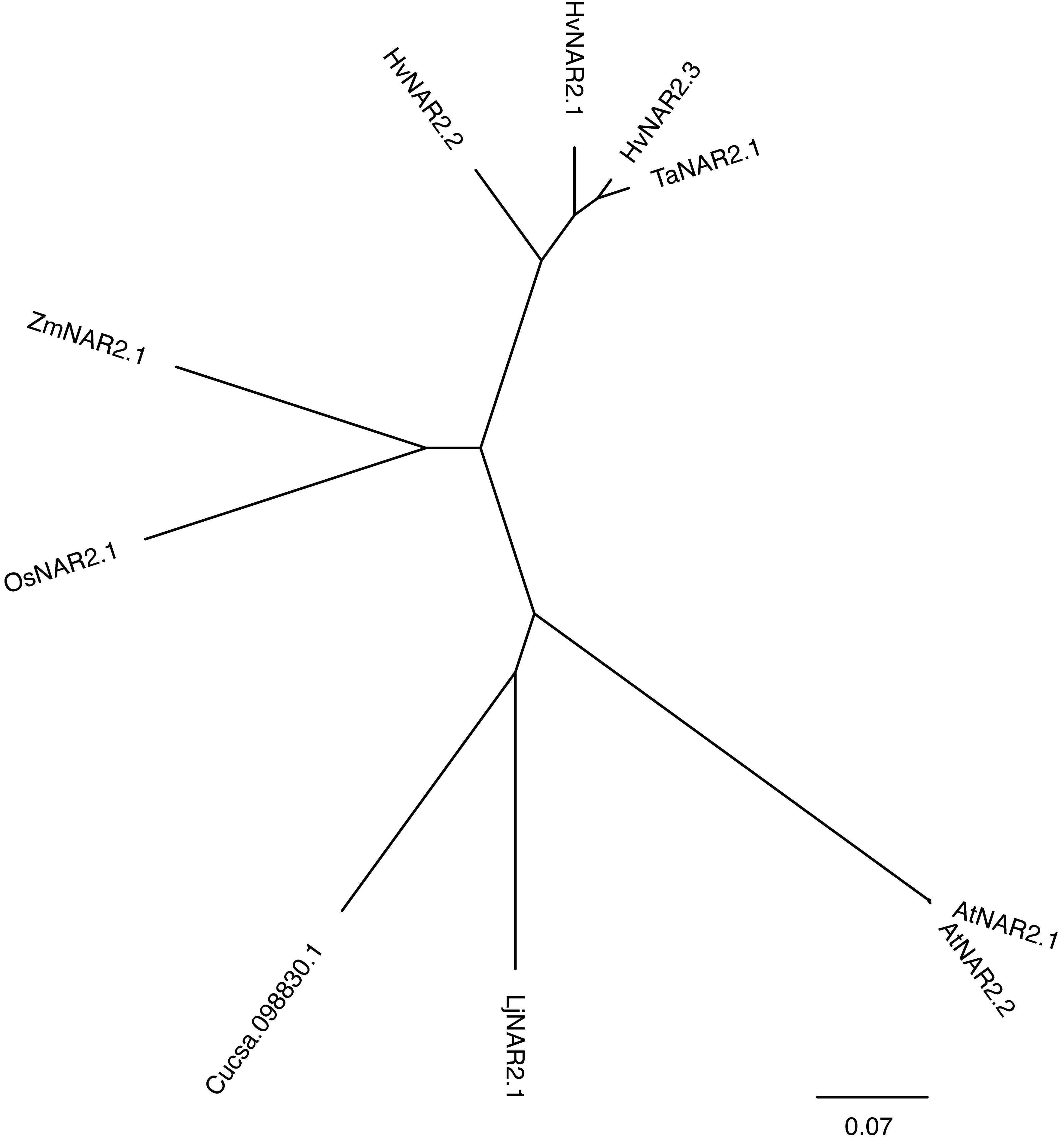

Supplement: Supplementary file 4 [file Image_3.tif]

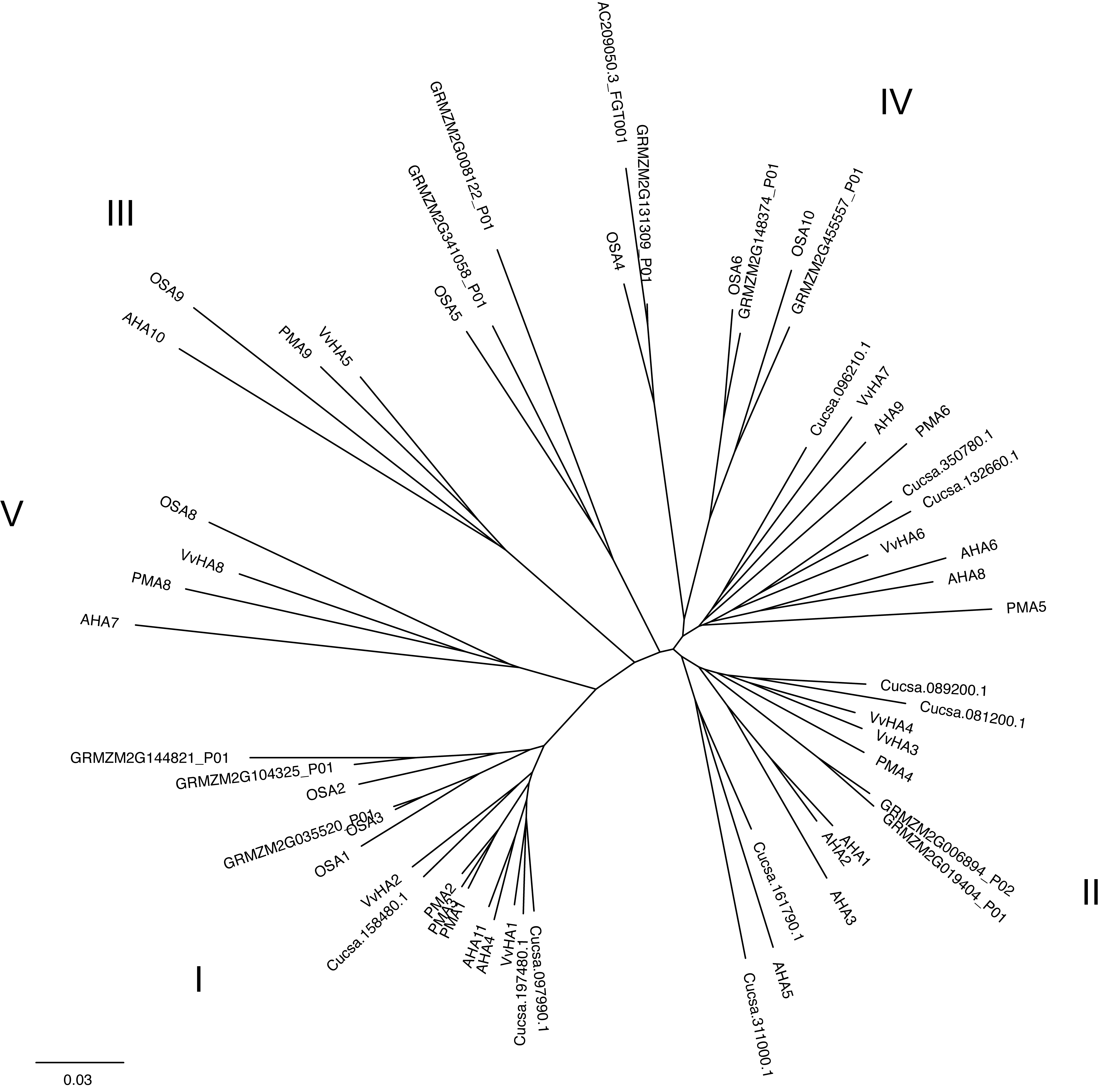

Supplement: Supplementary file 5 [file Image_4.tif]
