# Supplementary material for: Copper toxicity compromises root acquisition of nitrate in the high affinity range
Source: Front Plant Sci. 2023 Jan 20;13:1034425. doi: 10.3389/fpls.2022.1034425 (PMC9895927; doi:10.3389/fpls.2022.1034425)
Supplement: Supplementary file 6 [file Table_1.docx]

**Supplementary Table 1. Sequences of gene specific primers used for the quantitative RT-PCR analyses.**

| Gene | Forward (5’ – 3’) | Reverse (5’ – 3’) |
| --- | --- | --- |
| *CsNRT2.1* | AGGTTTGCATCAACAAAGTATTA | TCAAACATGAGTAGGAGTTGTA |
| *CsNRT2.3* | AGGCTATCATATGGGAAGCC | TTAAACATGAGGTGGGGAATC |
| *CsNRT3.1* | GTTAAGAAAGGTGGGAACTTG | GAAACTCATAAAGTCTATTCCTG |
| *CsHA2 (Cucsa.089200.1)* | GAAAAGTATTATTGCTTCCTCAT | TGTTGTCTCTTTCTCAAACATTT |
| *Cucsa.161790.1* | GTTGGGGTGTGGGTGCAG | CTGTATAGTGTTGTTGGATTGT |
| *CsHA3 (Cucsa.081200.1)* | CTGCTACAAAAAGTTGTTGCAG | CTCCTTAGTTTCTTTAGATTGG |
| *Cucsa.311000.1* | CGTTTTCGTTTAGGCTTCGTG | TTAGACTGTATAATGTTGATTAATAG |
